# Ganglioneuroma has a potential for lymph node metastasis, not impacting recurrence

**DOI:** 10.3389/fonc.2025.1632294

**Published:** 2026-01-05

**Authors:** Ender Eren Ozcelik, Ahmet Bilgehan Sahin, Yagmur Karaman, Buket Erkan Ozmarasali, Ulviye Yalcinkaya, Erdem Cubukcu, Adem Deligonul, Turkkan Evrensel

**Affiliations:** 1 Department of Medical Oncology, School of Medicine, Bursa Uludag University, Bursa, Türkiye; 2 Department of Pathology, School of Medicine, Bursa Uludag University, Bursa, Türkiye

**Keywords:** ganglioneuroma, lymph node, metastasis, neuroblastic tumor, recurrence

## Abstract

Ganglioneuromas are rare, benign tumors arising from neuroblastic cells in the autonomic sympathetic nervous system. While generally considered indolent, limited case reports suggest their potential for regional metastasis. This retrospective study analyzed the clinical, demographic, and pathological features of 25 adult patients diagnosed with ganglioneuroma at Bursa Uludag University, Faculty of Medicine, between April 2007 and November 2023. The cohort comprised 18 females (72%) and seven males (28%), with a median age of 42 years (range: 19–79). Tumors were most commonly located in the abdomen (64%), followed by the thoracic (24%) and head and neck regions (12%), with the adrenal gland being the primary site in 32% of cases. Symptoms were present in 56% of patients, including pain, vision loss, hypertension, and palpable masses, while the remaining were asymptomatic. Surgical resection was performed in 92% of cases, with a median tumor size of 7.5 cm (1.5–18 cm). Median follow-up time was 88.3(16.2 - 217.8) months. Regional lymph node metastases were identified in 8% of patients, but no distant metastases or recurrences were observed during follow-up. These findings, including the novel observation of regional metastases, contribute valuable insights to the limited literature on ganglioneuromas. Despite its benign nature, this study highlights the potential for lymph node metastasis. However, the relationship between lymph node metastasis and recurrence has not been documented. In this context, further research is essential to better understand the risk factors, tumorigenesis, and the optimal management strategies for this rare tumor.

## Introduction

Ganglioneuroma is a benign neuroblastic tumor originating from the autonomic sympathetic nervous system (central or peripheral). It can, therefore, develop in locations where autonomic ganglia are present, such as the adrenal glands, retroperitoneum, mediastinum/thoracic region, and cervical area (1, 2). Ganglioneuroma is considered the least aggressive among all neuroblastic tumors, as it consists exclusively of mature cells (3). It is also the rarest tumor in this category (3). Approximately one-third of ganglioneuromas are located in the adrenal region (4). Despite their slow-growing nature and benign histology, they have the potential to reach considerable sizes before detection. These tumors are most frequently diagnosed in young to middle-aged adults, with a notable female predominance (5).

Ganglioneuroma is usually diagnosed during examinations due to pain caused by the mass effect. A significant portion of patients are asymptomatic and can be detected incidentally. In cases where a definitive diagnosis of ganglioneuroma cannot be established without histological confirmation, surgical intervention becomes necessary, particularly if the tumor is large or presents with local compressive symptoms. The prognosis is generally excellent (6).

Due to its rarity, the literature on ganglioneuroma is limited, primarily to studies with small patient cohorts. Although considered benign, there is inadequate evidence regarding its metastatic potential, apart from a few published case reports suggesting this possibility (7, 8). In previous studies, data regarding the management and prognostic significance of lymphatic metastases have remained insufficient.

In this study, we aim to evaluate the characteristics of patients treated at our center, emphasizing similarities and differences compared to previously reported findings. In addition, we planned to present the clinicopathological features of the specific condition of lymph node metastasis and its impact on survival.

## Methods

### Patients

Clinical data were collected retrospectively from the electronic medical records of patients who admitted to Bursa Uludag University, Faculty of Medicine. The study included patients who had been histopathologically diagnosed with ganglioneuroma between January 2007 and December 2023 and were over 18 years old at the time of diagnosis. Patient demographics (age, sex), clinical presentations, tumor size, location, symptoms, surgical procedures, histopathological reports and follow-up records were systematically extracted and analyzed.

### Surgical procedures

All surgical resections were performed by specialized surgical teams after multidisciplinary evaluation. Standardized surgical procedures included complete excision of the tumor mass, along with regional lymph node dissection in cases where lymphatic involvement was suspected preoperatively or identified intraoperatively.

### Pathological evaluation

All pathological specimens were reviewed retrospectively by an experienced pathologist specialized in soft tissue tumors and sarcoma pathology. Hematoxylin and eosin (H&E) staining was routinely applied to all samples. Immunohistochemical staining was performed using markers including Synaptophysin, S-100 protein, Chromogranin A, and Neurofilament protein to confirm the neurogenic origin and maturity of the tumor cells. Slides were systematically evaluated for tumor differentiation, cellular composition, presence of atypia, and metastatic involvement.

### Ethical statement

This study was approved by the Ethics Committee of Bursa Uludag University (Decision Number: 2025/3/3). Due to its retrospective design, the ethical committee has waived subjects’ informed consent.

## Results

**Figure 1:**
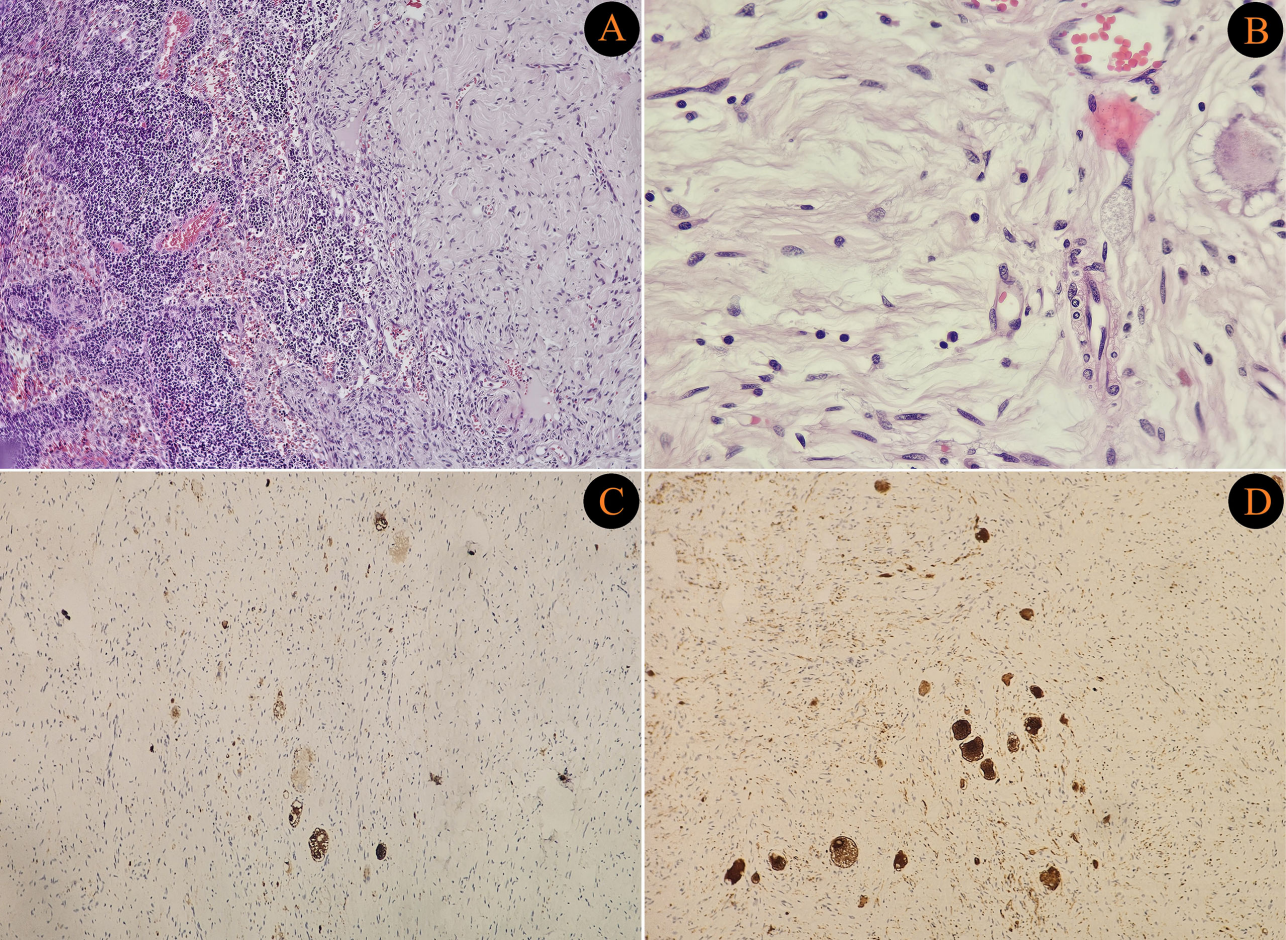
**(A)** Low-power view (H&E, ×40) showing adrenal ganglioneuroma with adjacent normal lymph node architecture on the left and tumor tissue on the right. **(B)** High-power view (H&E, ×100) demonstrating spindle-shaped Schwann cells with elongated nuclei and mature ganglion cells embedded in loose, fibrillary, homogeneous eosinophilic stroma. **(C)** High-power immunohistochemistry (Synaptophysin, ×100) revealing diffuse cytoplasmic positivity in ganglion cells. **(D)** High-power immunohistochemistry (Neurofilament, ×100) demonstrating strong positivity in ganglion cells.

**Figure 2:**
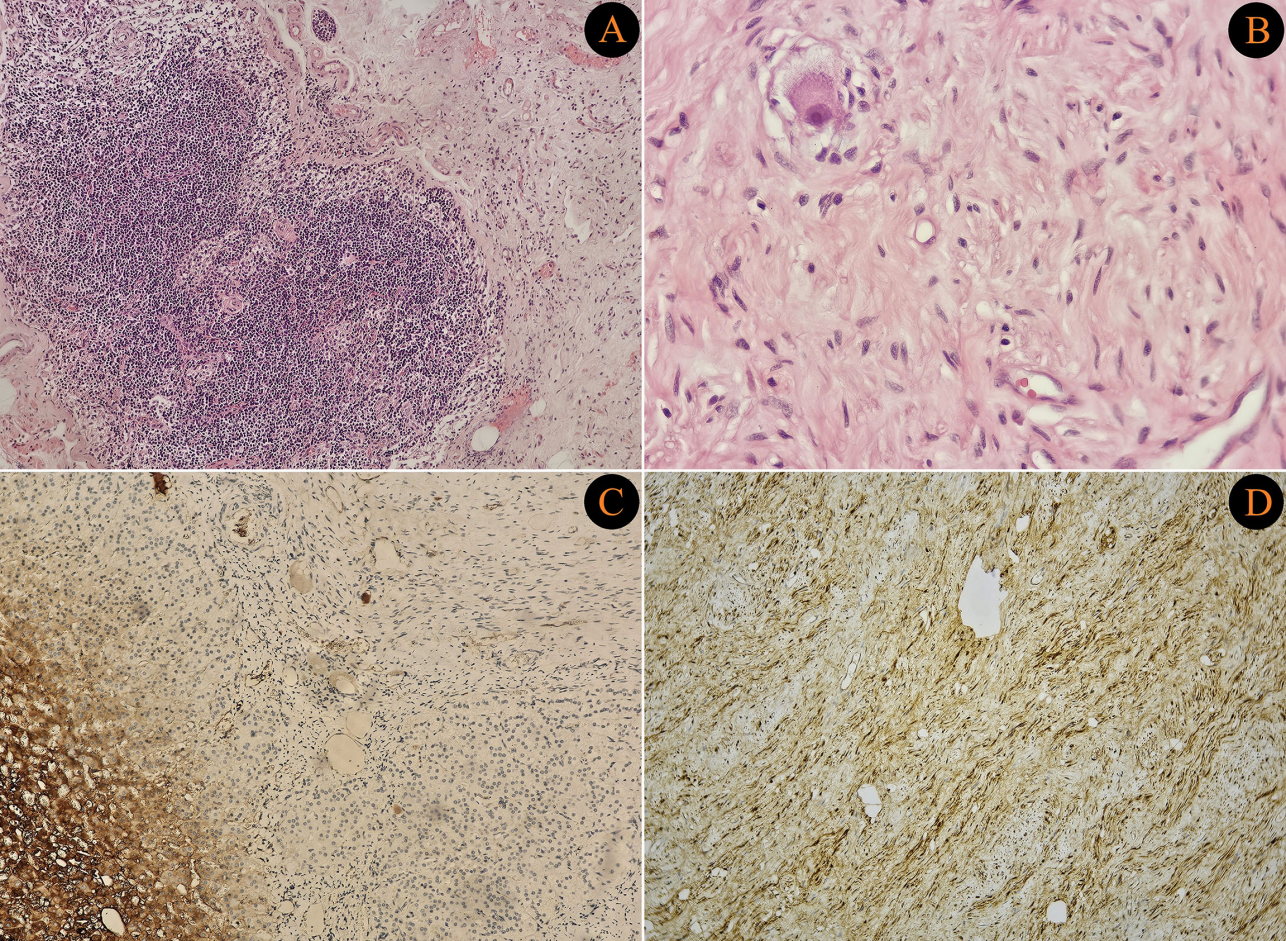
**(A)** Low-power view (H&E, ×40) of ganglioneuroma infiltrating a lymph node. **(B)** High-power view (H&E, ×100) showing spindle cells and ganglion cells arranged in fascicles within a fibrillary stroma. **(C)** High-power immunohistochemistry (Chromogranin, ×100) showing strong cytoplasmic positivity in ganglion cells. **(D)** High-power immunohistochemistry (S-100 protein, ×100) demonstrating diffuse positivity in tumor cells.

The demographic and clinical characteristics of the 25 patients and clinicopathological outcomes are demonstrated in **Table 1**. A total of 25 adult patients diagnosed with ganglioneuroma were retrospectively analyzed. Of the patients, 72% were female. The median age at diagnosis was 42 years (range: 19–79). Regarding tumor locations, 64% of patients had abdominal ganglioneuromas, 24% had tumors originating from the thoracic region, and three patients had tumors in the head and neck region. Across all groups, the most common primary tumor location was the adrenal gland, observed in 8 patients. Clinical presentations varied: 44% of the patients presented with pain, one patient with vision loss, one patient with hypertension, and one patient with a palpable mass, while 44% of the patients were asymptomatic and diagnosed incidentally. **Figures 1**, **2** demonstrate pathological images of the cases with lymph node metastasis.

**Table 1:** Demographic, clinicopathological features and outcomes of patients with ganglioneuroma.

Parameter	n	%
Age at Diagnosis (years)	42 (19-79) Median (range)	
Sex		
• Female • Male	18 7	72.0 28.0
Tumor Location		
• Abdomen • Thoracic • Head and Neck • Adrenal (within abdominal group)	16 6 3 8	64.0 24.0 12.0 32.0
Symptomatic		
• Pain • Vision Loss • Hypertension • Palpable Mass	14 11 1 1 1	56.0 44.0 4.0 4.0 4.0
Asymptomatic	11	44.0
Tumor Size (cm)	7,5 (1,5-18) Median (range)	
Surgical Resection		
• Performed • Not Performed (Biopsy only)	23 2	92.0 8.0
Lymph Node Metastasis		
• Present • Absent	2 23	8.0 92.0
Distant Metastasis	None	0.0
Recurrence During Follow-up	None	0.0

Of the patients, 92% underwent surgical resection, while two patients had no surgery after diagnosis was made through biopsy. The median tumor size for the entire cohort was 7.5 cm (range: 1.5–18 cm). Of these, complete resection (R0) was achieved in all cases. Median follow-up time was 88.3 (16.2 - 217.8) months. No progression was observed during follow-up in the two patients who were diagnosed by biopsy without surgical resection.

Regional lymph node metastases were detected in two female patients (8%). No distant metastases were observed, and no recurrences were detected during follow-up. The first patient was a 30-year-old female with a 5.5 cm tumor located in the left adrenal gland. Histopathological analysis revealed 5 out of 12 dissected lymph nodes to be positive for metastasis. The second patient was a 60-year-old female with a 9 cm tumor located in the para-aortic region at the level of the renal hilum. All 4 of the resected lymph nodes were positive for ganglioneuroma metastasis.

## Discussion

Our study stands as one of the largest single-center investigations in the literature, providing valuable clinical data on this rare entity. We demonstrate that, although ganglioneuromas are benign tumors, they have the potential to metastasize to regional lymph nodes. Notably, our findings indicate that 8% of the patient cohort showed evidence of regional lymph node metastasis, marking this as the first research article to present such data.

Lymph node involvement in ganglioneuromas remains an area of uncertainty in the current literature. This ambiguity necessitates a clearer definition of the histopathological evaluation criteria and classification of the tumor. Furthermore, it raises the possibility of underlying heterogeneity, including features such as mixed-type or borderline components. Possible explanations for lymph node metastasis include the maturation of initially immature tumors, such as neuroblastomas, into more differentiated forms over time, with estrogen considered as a potential contributing factor (9), or the presence of minimal immature cellular components due to histological heterogeneity within some ganglioneuromas. These scenarios may account for the rare reports of nodal involvement. Also rare reports have suggested a potential for malignant transformation, particularly in the setting of neurofibromatosis type 1 (10).

In our study, no immature or mixed form cells were observed in the pathology slides of patients with lymph node metastasis. The fact that both patients with lymph node metastasis were female supports the literature suggesting estrogen’s role in the maturation of neuroblastic tumors. In the future, a revised pathological classification may be necessary to clarify this entity more definitively. Therefore, we believe that both the histopathological examination criteria of the primary tumor and the WHO classification need to be updated. Future studies might consider employing extensive histological sampling and molecular techniques to detect minimal immature cellular components.

The majority of the literature reports ganglioneuromas as benign entities with a favorable prognosis. In studies involving larger cohorts, no recurrence or metastasis has been reported, except in cases associated with non-neuroblastic composite ganglioneuromas or other neoplasms (5). While it has been proposed that ganglioneuromas may recur as more aggressive tumors such as rhabdomyosarcoma, there is no definitive evidence regarding the trustworthy source of such recurrences (11, 12). Rare case reports have documented the metastatic potential of ganglioneuromas (7, 8).

Our finding of an 8% incidence of regional lymph node metastasis contrasts with the widely accepted benign nature of ganglioneuromas reported in previous literature. Prior case series (13, 14) reported negligible rates of metastatic spread, emphasizing the novelty of our observation. There is a lot of previously documented isolated case reports of lymph node involvement (8, 15, 16); however, our series constitutes the largest single-center report highlighting regional lymphatic involvement.

In case series reported in the literature, the proportion of symptomatic patients varies between 17.1% and 73.4% (13, 14). The most frequently reported complaints in both datasets are size-related symptoms such as pain and palpable masses. Our findings’ frequency and symptom characteristics are consistent with those of the existing literature.

The largest meta-analysis of ganglioneuroma to date, encompassing all age groups, was published in 2021. In this study, 364 cases were identified, with 65.7% occurring in adults, and a female predominance of 62% was noted. Incidentally detected tumors accounted for 24.5% of cases. The most commonly affected sites were the abdomen/pelvis (66.2%), followed by adrenal ganglioneuromas (32.1%). No recurrences were reported, confirming the benign behavior of these tumors irrespective of age (5). Similarly, our study showed a female predominance of 72% compared to 28% in males. We found that 64% of cases were abdominal, with half being adrenal.

Several unresolved issues remain in the field of ganglioneuroma research. One such issue is the lack of clarity regarding risk factors and the environmental contributors to tumorigenesis (17). Additionally, due to the rarity of this tumor and the heterogeneity of statistical evidence, there is a need to develop scoring systems for optimal management (18). Another debate concerns the necessity of tumor resection, as removal may not always be required, except in symptomatic cases or when imaging shows a certain rate of tumor growth. However, the possibility of high malignancy cannot be excluded until a complete histological evaluation and profiling of the lesion is performed. Therefore, differential diagnosis after surgery is crucial, especially in cases of adrenal ganglioneuromas (19).

Our study has several limitations, primarily its retrospective and single-center design, small sample size and absence of prospective standardized lymphatic evaluation protocols. In addition, molecular analyses such as genomic profiling or phenotypic characterization (e.g., gene expression or DNA methylation studies) could not be performed. Although a limited number of patients, our study, to our knowledge, is the largest single-center experience reporting the clinical course of such a rare disease, so we think it contributes to the literature.

These findings highlight the need for refinement in both the pathological classification and in the biological understanding and behavior of ganglioneuroma. Future research should involve multicenter, prospective studies with centralized pathological review to confirm our findings and clarify the clinical significance of lymph node involvement. Incorporating molecular profiling methods—such as DNA methylation analysis, gene expression subtyping, and hormonal receptor assessment (e.g., estrogen or NGF signaling pathways)—may help determine whether nodal involvement represents true metastatic behavior or a variant of multifocal maturation. DNA methylation–based tumor classification has already demonstrated clinical utility in other tumor types, such as central nervous system neoplasms (20), and similar approaches may prove valuable in characterizing biological subtypes of ganglioneuroma. Identification of predictive biomarkers could also guide individualized clinical decision-making, particularly regarding the necessity and extent of regional lymphadenectomy in selected patients.

In conclusion, while ganglioneuroma is widely recognized as a benign tumor, regional lymph node metastases may be observed in up to 10%, although it does not impact survival. Further pathological evaluation and refinement of classification criteria may be required to better elucidate the true biological behavior of this entity.

## Data Availability

The original contributions presented in the study are included in the article/Supplementary Material. Further inquiries can be directed to the corresponding author/s.
